# Monoclonal Antibodies Targeting the Alpha-Exosite of Botulinum Neurotoxin Serotype/A Inhibit Catalytic Activity

**DOI:** 10.1371/journal.pone.0135306

**Published:** 2015-08-14

**Authors:** Yongfeng Fan, Isin N. Geren, Jianbo Dong, Jianlong Lou, Weihua Wen, Fraser Conrad, Theresa J. Smith, Leonard A. Smith, Mengfei Ho, Melissa Pires-Alves, Brenda A. Wilson, James D. Marks

**Affiliations:** 1 Department of Anesthesia, University of California, San Francisco, San Francisco, California, United States of America; 2 Molecular and Translational Sciences Division, United States Army Medical Institute of Infectious Diseases, Fort Detrick, Maryland, United States of America; 3 Medical Countermeasures Technology, United States Army Medical Research and Material Command, United States Army Medical Institute of Infectious Diseases, Fort Detrick, Maryland, United States of America; 4 Department of Microbiology, University of Illinois at Urbana-Champaign, Urbana, Illinois, United States of America; Institute Pasteur, FRANCE

## Abstract

The paralytic disease botulism is caused by botulinum neurotoxins (BoNT), multi-domain proteins containing a zinc endopeptidase that cleaves the cognate SNARE protein, thereby blocking acetylcholine neurotransmitter release. Antitoxins currently used to treat botulism neutralize circulating BoNT but cannot enter, bind to or neutralize BoNT that has already entered the neuron. The light chain endopeptidase domain (LC) of BoNT serotype A (BoNT/A) was targeted for generation of monoclonal antibodies (mAbs) that could reverse paralysis resulting from intoxication by BoNT/A. Single-chain variable fragment (scFv) libraries from immunized humans and mice were displayed on the surface of yeast, and 19 BoNT/A LC-specific mAbs were isolated by using fluorescence-activated cell sorting (FACS). Affinities of the mAbs for BoNT/A LC ranged from a K_D_ value of 9.0×10^−11^ M to 3.53×10^−8^ M (mean K_D_ 5.38×10^−9^ M and median K_D_ 1.53×10^−9^ M), as determined by flow cytometry analysis. Eleven mAbs inhibited BoNT/A LC catalytic activity with IC_50_ values ranging from 8.3 ~73×10^−9^ M. The fine epitopes of selected mAbs were also mapped by alanine-scanning mutagenesis, revealing that the inhibitory mAbs bound the α-exosite region remote from the BoNT/A LC catalytic center. The results provide mAbs that could prove useful for intracellular reversal of paralysis post-intoxication and further define epitopes that could be targeted by small molecule inhibitors.

## Introduction

Botulism is caused by botulinum neurotoxins (BoNTs), produced by the bacterium *Clostridium botulinum*. BoNTs are the most lethal substances known. Human LD_50_ values have been estimated to be ~ 10 ng/kg via inhalation and ~ 1 **μ**g/kg via ingestion [1,2]. As a result, BoNTs are classified by the Centers for Disease Control and Prevention (CDC) as among the highest risk threat agents for bioterrorism (Tier 1 Category A agents) [1,3]. Seven or eight serotypes of BoNTs have been described (A-H), at least four of which have been reported to cause human botulism (BoNT/A, B, E, F) [4–7]. BoNTs are 150-kDa proteins composed of three functional domains [8], a binding domain (H_C_), translocation domain (H_N_) and catalytic domain (LC). The H_C_ binds carbohydrate and protein receptors on the presynaptic membrane [9–11] leading to BoNT endocytosis. Once endocytosed, the H_N_ forms a channel across the endosomal membrane allowing delivery of the LC into the cytoplasm [12–15]. The LC is a zinc-dependent endopeptidase that cleaves one or more Soluble NSF Attachment Protein Receptor (SNARE) proteins depending on the BoNT serotype. For BoNT/A, the SNARE substrate is synaptosome-associated protein of 25,000 Daltons (SNAP25), whose cleavage blocks synaptic vesicle fusion and acetylcholine neurotransmitter release [16]. SNAP25 binds BoNT/A LC in an extended cleft resulting in cleavage at position Gln197-Arg198 [17,18]. Prior to neuronal entry, the catalytic cleft of the holotoxin is occupied by a portion of the H_N_ called the “belt” [19].

The multi-domain structure of BoNTs provides a number of ways to prevent or treat botulism. The current mainstay of treatment for botulism is antitoxin [20]. Antitoxins, such as equine antitoxin and human botulism immunoglobulin, are used to treat adult [21,22] and infant botulism [23], respectively. Antitoxin works by clearing BoNT from the circulation before it can accumulate inside the neuron [24] and by blocking BoNT entry into neurons by binding to the H_C_ [25]. Antibody may also be able to inhibit translocation by binding to the H_N_ and/or LC extracellularly, presumably by piggybacking into the neuron attached to the BoNT and subsequently interfering with the function of these domains [15,26]. Identification of the protein and ganglioside receptor-binding sites on the BoNT H_C_ may also permit the design of small molecule drugs that can block toxin binding and uptake [27–29].

A limitation of the above therapeutics is that they do not work once the toxin has entered the cytosol of the neuron and therefore cannot be used to reverse paralysis. Thus, there is now considerable interest in developing inhibitors of the H_N_ and LC domains, which can be coupled with alternative delivery vehicles for transport of the inhibitor cargo across target cell membranes [30–33]. Since the window to prevent translocation is relatively short, most attention has been focused on small molecule or peptide-based inhibitors that prevent the catalytic domain from cleaving their respective SNARE substrates. Such inhibitors typically mimic the target substrate and bind in or around the substrate cleavage pocket [34,35]. The crystal structure of the substrate SNAP25 complexed to the BoNT/A LC showed the extended nature of ligand recognition and identified potential exosites of substrate binding that are remote from the catalytic active site [36]. Such exosites have been targeted for inhibitor development against other toxins [37,38]. Alternatively, the active sites or exosites could be targeted for binding by antibodies for inhibition of toxin activity. We previously reported the isolation of a single-domain camelid VHH antibody that bound the BoNT/A LC α-exosite with a K_D_ of 147 pM and potently inhibited SNAP25 cleavage [39].

To further explore the range of binding sites for potential BoNT/A LC-specific antibody-based inhibitors, mice were immunized with BoNT/A and BoNT/A LC, and repertoire cloning and yeast display were used to isolate BoNT/A LC- binding mAbs. By determining which mAbs inhibited SNAP25 cleavage and identifying their fine epitopes, the range of epitopes associated with inhibition of catalysis was further defined.

## Results and Discussion

### Construction of a single-chain Fv yeast display vector

**Fig 1:**
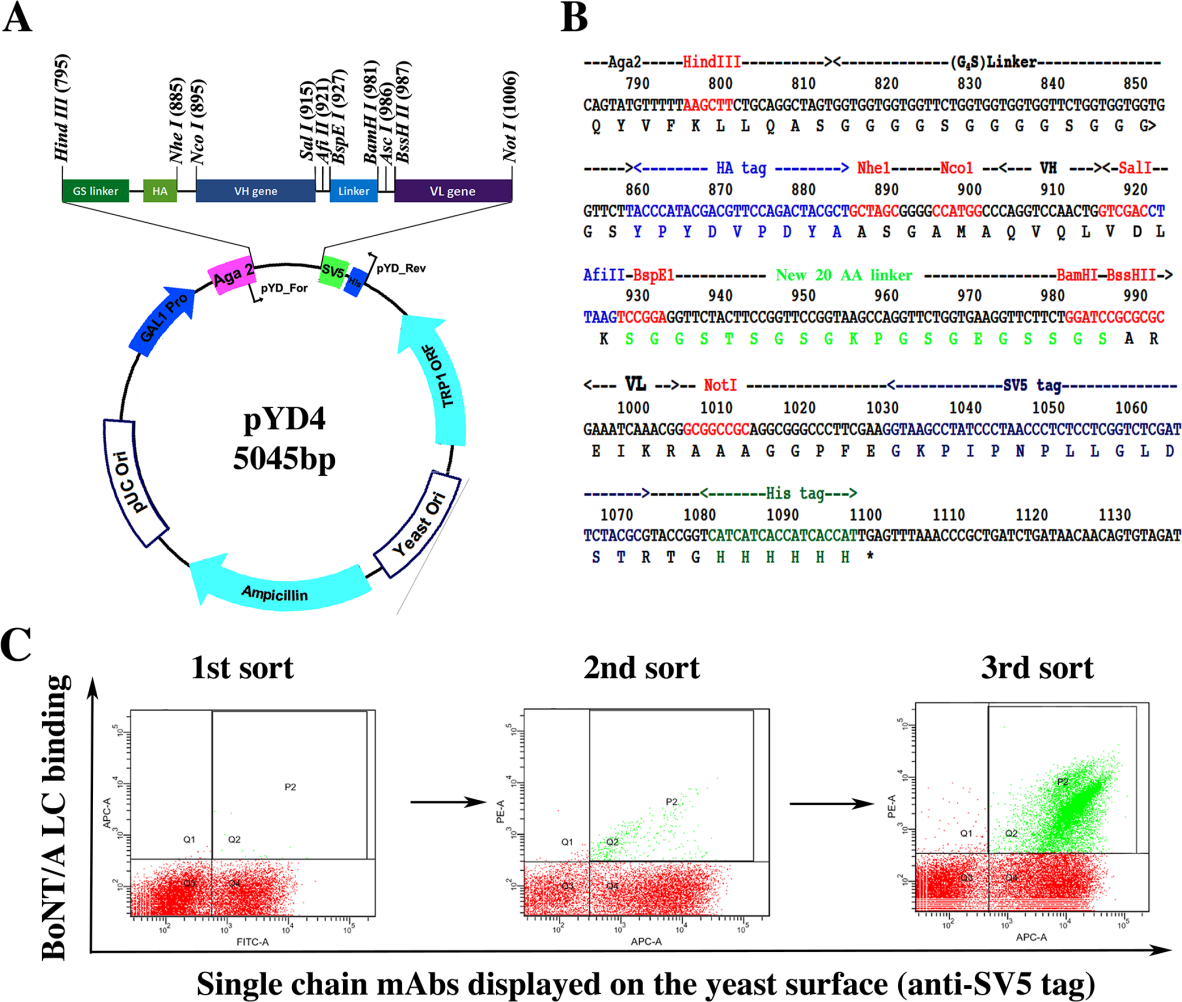
The modified plasmid pYD4 and the library sorting strategy. **A.** Map of pYD4 plasmid. **B**. The poly linker of pYD4. Compared with the yeast display vector pYD2, a new linker with 20 amino acids different from the (G_4_S)_3_ linker upstream of the scFv gene was built in the plasmid pYD4, flanked by restriction enzyme sites for cloning of V_H_ or V_L_ genes individually, rather than as a scFv. An HA tag was introduced between the (G_4_S)_3_ linker and scFv as an alternative detection marker besides the SV5 tag downstream of the final scFv construct. **C.** Dot plots of the results of a typical three-round FACS sorting of a scFv library constructed in pYD4. For each round of FACS sorting, fluorescence-conjugated secondary antibody bound to BoNT/A LC (50 nM) was used to stain yeast libraries BoNT/A LC binding is shown on the Y-axis and the scFv display level on the X-axis after staining with anti-SV5 tag antibody. The yeast population with BoNT/A LC bound was collected by using the indicated gate settings shown for the yeast labeled green in the dot plots.

The yeast display vector pYD2 [40] was modified to allow single-chain Fv (scFv) library construction by sequential cloning of immunoglobulin heavy (V_H_) and light (V_L_) chain gene repertoires (Fig 1A). The resulting vector (pYD4) used the linker (SGGSTSGSGKPGSGEGSSGS) between the V_H_ and V_L_ genes (kappa and lambda chain variable regions, V**κ** and V_**λ**_), which were flanked by restriction enzyme sites for cloning the V-genes. The SV5-tag and His_6_-tag were retained at the C-terminal of scFv in pYD4 and an additional HA tag was introduced downstream of the (G_4_S)_3_ linker and at the N-terminal of scFv as an alternative scFv expression marker. Finally, the modified poly-linker (Fig 1B) was synthesized and cloned into the pYD2 backbone for scFv library construction and affinity maturation for selected clones. The V_L_ genes were first cloned into pYD4, then the V_L_ library prepped and the V_H_ gene repertoire inserted by using gap repair homologous recombination to obtain the scFv library. Advantages of pYD4 over pYD2 include elimination of the polymerase chain reaction (PCR) splicing reaction, reduced risk of loss of diversity of the library due to the need to conduct several rounds of PCR amplification of V genes, and simpler light or heavy chain shuffling for affinity maturation of isolates.

### Library construction and mAb isolation/characterization

Spleens from female CD-1 mice (3–4 for each antigen) immunized with BoNT/A1 LC, BoNT/A1 LC-belt or BoNT/A1 LC-H_N_ and peripheral blood from 12 adult human donors immunized with BoNT/A, B, C, D and E pentavalent toxoid were used for scFv library construction (Table 1). Human donors were laboratory workers being immunized to work with BoNT who were recruited via an informational letter and who signed informed consent under a protocol approved by the University of California, San Francisco Institutional Review Board. For immunized mouse libraries, V**κ** gene repertoires amplified from spleen were cloned into pYD4 to create V**κ** libraries. The V_H_ gene repertoire from the immunized mice were cloned into the V_K_ libraries to create scFv libraries. The human donor libraries were constructed by using pYD2 and the PCR assembled scFv repertoire cloned as previously described [41]. The size of the scFv libraries was between 1x10^7^ and 1x10^8^ yeast transformants. The BoNT/A1 LC fragment was used for isolation of BoNT/A LC specific mAbs by FACS sorting [40]. After three rounds of sorting, individual colonies were picked and characterized for BoNT/A LC binding, yielding 19 unique mAbs as determined by DNA sequencing of the scFv genes (17 from the murine libraries and two from the human libraries) (Table 2). Further scFv analysis by flow cytometry showed that murine scFv 9B2, 10C9, 10F9 10B4, 7C8, 12A11 and human scFv ING2 and 5A20.4 bound both BoNT/A LC and BoNT/A holotoxin. The remaining scFv bound BoNT/A LC fragment but not bind BoNT/A holotoxin.

**Table 1:** Yeast display libraries constructed and used for BoNT/A LC mAb generation.

Library source	Immunizing Antigen	Library size
Mouse 3	BoNT/A LC	4 x 10^7^
Mouse 4	BoNT/A LC-Belt	1 x 10^8^
Mouse 5	BoNT/A LC-H_N_	1 x 10^8^
Human Donor 6	BoNT/A, B, C, D, E toxoid	~10^7^
Human Donor 10	BoNT/A, B, C, D, E toxoid	~10^7^

**Table 2:** Binding and endopeptidase inhibition characteristics of BoNT/A LC specific scFv mAbs.

Clone or source	Yeast displayed scFv K_D_ (×10^-9^M)^1^ (± Standard Deviation)			Endopeptidase inhibition ^2^	Epitope recognized^3^
	BoNT/A LC	BoNT/A1 holotoxin	BoNT/A2 holotoxin		
*Murine scFv*					
1C7	34.7(±5.90)	NB^4^	NB	No	I
1C10	4.85 (±0.66)	NB	NB	Yes	I
1D8	3.35 (±1.48)	NB	NB	Yes	I
1D9	9.13 (±2.47)	NB	NB	No	I
1G11	0.114 (±0.05)	NB	NB	Yes	I
1H5	10.62 (±2.33)	NB	NB	Yes	I
9B2	0.67 (±0.18)	>200	>100	No	I
10C9	0.36 (±0.12)	>500	>100	Yes	I
10H10	11.09 (±0.91)	NB	NB	Yes	I
10H11	0.92 (±0.47)	NB	NB	No	I
10B12	0.53 (±0.05)	NB	>100	Yes	I
10F9	0.09 (±0.03)	>500	>500	Yes	I
11D8	0.22 (±0.10)	NB	NB	Yes	I
10B4	1.53 (±0.61)	>500	>200	No	I
1D2	10.86 (±1.05)	NB	NB	No	II
7C8	0.94 (±0.38)	0.47 (±0.08)	0.50 (±0.07)	Yes	III
12A11	25.8 (±3.98)	2.08 (±0.96)	1.76 (±0.23)	No	III
*Human scFv*					
ING2	16.28 (±1.35)	0.11 (±0.05)	0.12 (±0.04)	Yes	III
5A20.4	1.05 (±0.63)	0.18 (±0.08)	NB	No	IV

^1^ Affinities measured for yeast-displayed scFv using flow cytometry and the indicated antigen.

^2^ Inhibition of substrate cleavage determined by SDS-PAGE and FRET (Fig 2).

^3^ Epitope recognized by scFv arbitrarily grouped by number depending on competition for the same binding site illustrated in Fig 2.

^4^ NB; no binding of the antigen was detected at 200nM.

The affinity of the scFv on yeast was measured by flow cytometry, exhibiting a K_D_ for BoNT/A1 LC ranging from 9.0×10^−11^ M—3.53×10^−8^ M, with a mean K_D_ of 5.38 × 10^−9^ M. Eleven of the 19 scFvs did not have detectable binding to BoNT/A holotoxin at 200nM, four scFv (9B2, 10C9, 10F9, and 10B4) bound BoNT/A LC with higher affinity than BoNT/A holotoxin, three scFv (7C8, 12A11 and ING2) bound BoNT/A holotoxin with higher affinity than BoNT/A LC and one scFv (5A20.4) bound both with comparable affinity (Table 2).

Selected scFv mAbs were subcloned and expressed as IgG1 from CHO cells. Four scFv that bound BoNT/A LC but did not bind holotoxin (10B12, 10F9, 11D8 and 1D2) showed the same pattern of binding after conversion to IgG. The affinity of yeast-displayed scFv for binding to measured by flow cytometry (Table 3, K_D_ 1.1 × 10^−10^ M-> 5.0 × 10^−7^ M) is higher (lower affinity) than for IgG measured by KinExA (Table 4, K_D_ 4.2 × 10^−12^–5.12 × 10^−10^ M). This is likely accounted for by the greater stability of IgG compared to scFv and an apparent limit in the ability to measure very high affinity interactions by flow cytometry and is consistent with comparisons of affinity made for scFv vs. IgG for other antigens [40,42]. In contrast, except for 5A20.4, the affinity measured by KinExA for IgG binding to BoNT/A LC (> 1.0 x 10^−6^–8.57 × 10^−8^ M) were higher (lower affinity) than that measured for yeast-displayed scFv by flow cytometry (9.0×10^−11^–2.58 × 10^−8^ M) (Table 3). This difference in K_D_ values may have been due to conformational differences in LC under different buffer conditions or steric hindrance of the larger IgG in accessing specific epitopes resulting in a lower association rate constant.

**Table 3:** K_D_ values of IgGs measured using KinExA ^1^

Antibody	K_D_ (×10^-9^M)	K_D_ BoNT holotoxin		
	BoNT/A1 LC	A1	A2	A3
**7C8**	1.39 (1.62–1.18)	9.22×10^−3^ (11.00–7.62 × 10^−3^)	2.89 × 10^−3^ (4.99–0.99 × 10^−3^)	10.21 × 10^−3^ (13.84–7.09 × 10^−3^)
**10B12**	44.59 (<87.71)	NB	ND	ND
**10F9**	9.81 (<24.92)	NB	ND	ND
**11D8**	20.85 (30.11–13.99)	NB	ND	ND
**12A11**	>1000	10.21 × 10^−3^ (12.49–8.17 × 10^−3^)	7.34 × 10^−3^ (8.46–6.29×10^−3^)	4.20 × 10^−3^ (<12.45 × 10^−3^)
**1D2**	18.72 (26.39–11.79)	NB	NB	NB
**10B4**	9.84 (13.21–6.49)	0.51 (0.62–0.39)	ND	ND
**ING2**	115.43 (134.99–93.62)	17.07 × 10^−3^ (19.23–15.08 × 10^−3^)	14.24 × 10^−3^ (17.70–11.27×10^−3^)	NB
**5A20.4**	3.12 × 10^−3^ (4.19–2.24 × 10^−3^)	11.48 × 10^−3^ (18.01–6.59 ×10^−3^)	NB	NB

^1.^ Determined in a single measurement, 95% confidence intervals are shown in parentheses. ND: not determined, NB: no binding detectable.

**Fig 2:**
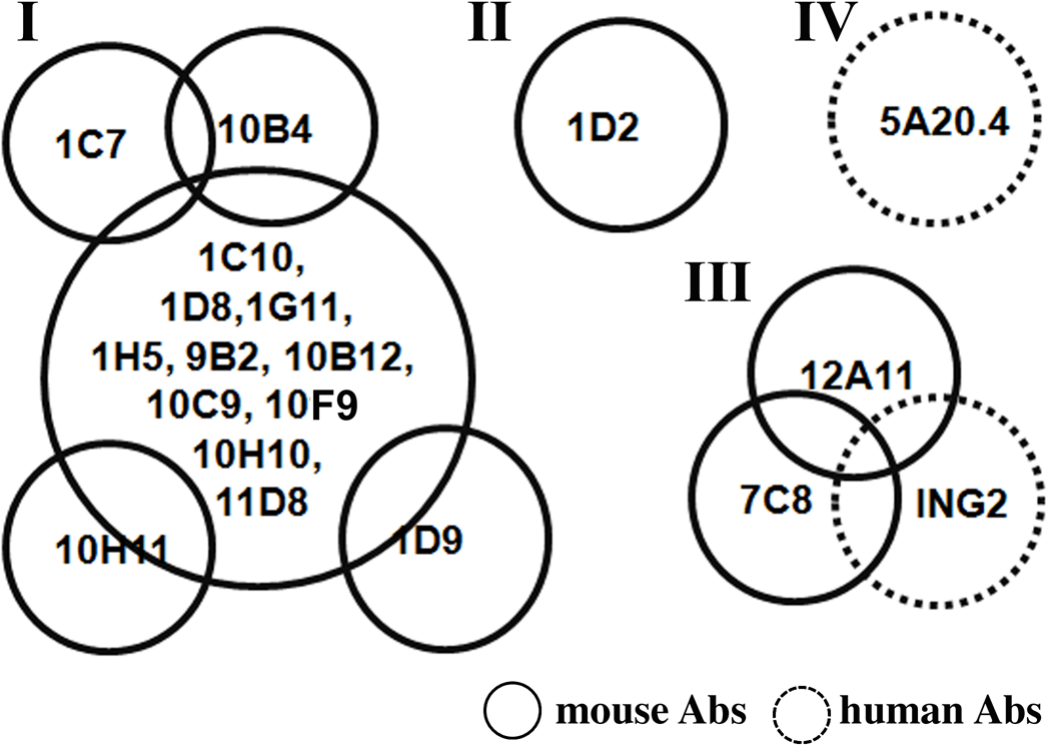
Classification of scFv epitopes on BoNT/A LC based on ability of scFv to bind simultaneously. Soluble BoNT/A LC was captured by yeast-displayed scFv and the ability of phage-displayed scFv to bind BoNT/A LC was determined by flow cytometry. The 19 scFv were clustered into 4 groups (I–IV), 14 of which bound epitope I.

Next, the epitopes recognized by each scFv were classified by determining the ability of mAbs to compete for binding to BoNT/A LC. In the assay, BoNT/A LC captured by yeast-displayed scFv was probed with phage-displayed scFvs, revealing at least four non-overlapping epitope clusters among the nineteen mAbs (Fig 2). Fourteen mAbs shared a single large overlapping epitope (Epitope group I). Among the 14 mAbs, 10H11, 1D9 and 1C7/10B4 did not affect the binding of each other but their binding was inhibited by other cluster I mAbs, suggesting that they had slightly different epitopes although they belonged to the same cluster. mAbs 1D2 and 5A20.4 bound unique epitopes (II and IV, respectively) while 12A11, 7C8 and ING2 all bound epitope cluster III.

### mAb inhibition of BoNT/A-LC cleavage of SNAP25

**Fig 3:**
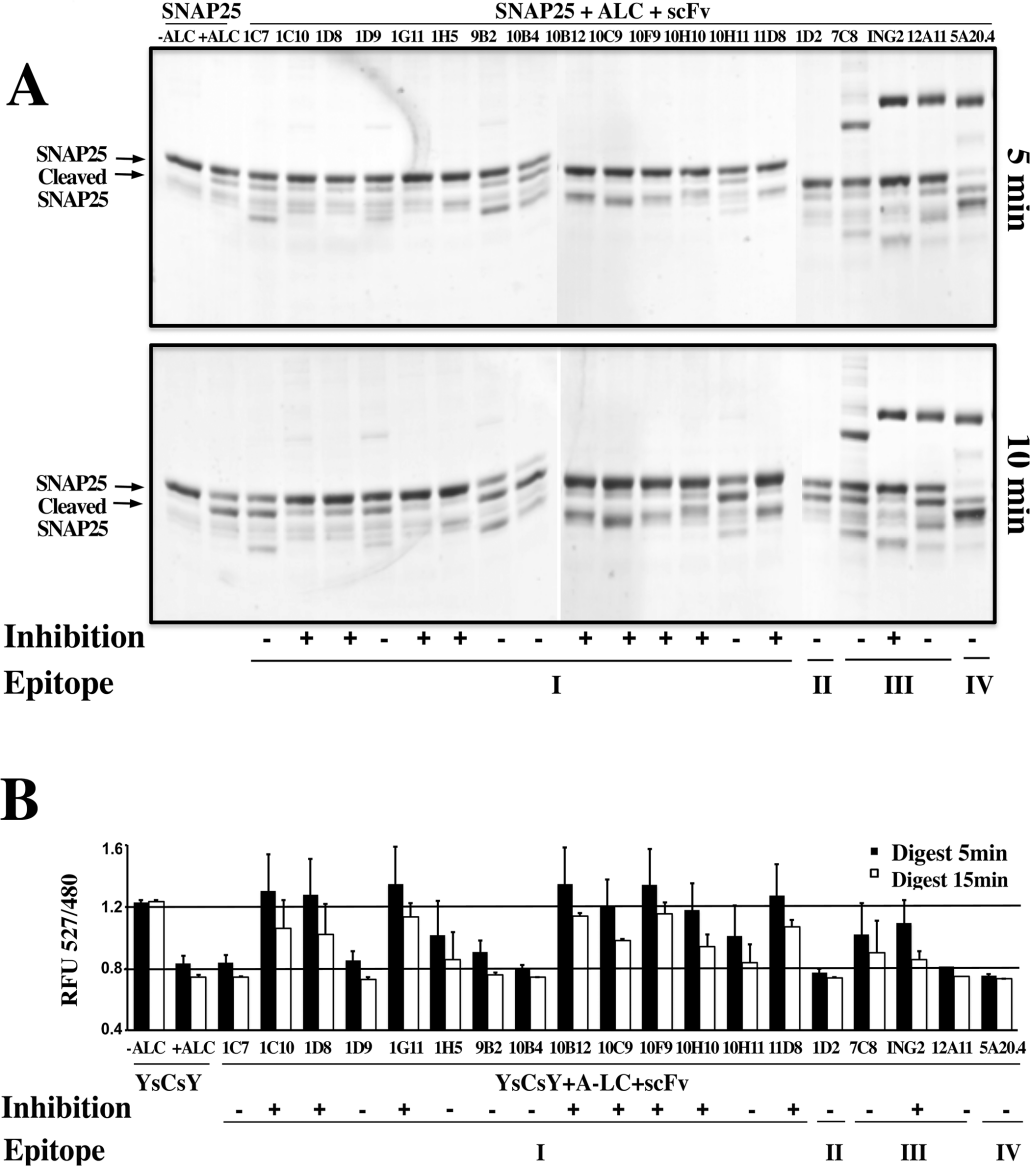
mAb inhibition of BoNT/A LC endopeptidase activity. **A**. SDS-PAGE-based substrate cleavage assay: BoNT/A LC (25 nM) and 20 molar excess of mAb were mixed in Tris buffer (50 mM, pH 8.0). GST-SNAP-25 (141–206) peptide substrate (5 μM) was added to initiate the reaction. Images of Coomassie-stained SDS-PAGE gels of the results after 5 min (the upper panel) or 15 min (lower panel) incubation with the intact substrate and cleaved product indicated by arrows. Ability to inhibit SNAP-25 cleavage was scored as positive (+) or negative (-). Additional bands in the SDS-PAGE gel likely represent GST-SNAP25 breakdown products or impurities from the mAbs. **B.** Plot of the results from the FRET screening assay for inhibition of substrate cleavage. The YsCsY substrate (2 μM) was mixed with each of the indicated mAbs (200 nM) and BoNT/A LC. (400 pM). The mean (± standard deviation) of the ratio of emissions at 527 nm to 480 nm after 5 min or 15 min are shown. Ratios >0.8 at 15 minutes were interpreted to indicate inhibition of BoNT/A-mediated cleavage by the mAb, denoted by (+). The epitope clusters (I-IV, Fig 2) are shown below each scFv. Nine mAbs in epitope cluster I (1C10, 1D8 1G11, 1H5, 10B12, 10C9, 10F9, 10H10 and 11D8) strongly inhibited BoNT/A LC cleavage.

To screen for mAbs that inhibited the proteolytic activity of BoNT/A LC, soluble scFvs were added to reaction mixtures and evaluated using SDS-PAGE and FRET methods. Soluble scFvs were expressed in *E*. *coli* and purified by IMAC to greater than 90% purity. For the SDS-PAGE based endopeptidase assay, the substrate GST-fused SNAP25 (141–206) was incubated with BoNT/A LC in 25nM Tris-Cl buffer for 5 minutes and 15 minutes, with or without addition of scFvs. The amount of intact GST-SNAP25 remaining as determined by SDS-PAGE indicated the degree of inhibition by mAbs (Fig 3A). We also used a FRET-based screen for scFv inhibition of BoNT/A LC cleavage of SNAP [43,44]. In this assay, the emission ratio at 527 nm and 480 nM (RFU527/480) reflects the degree of substrate (Yellow Fluorescent Protein(YFP)-SNAP25-Cyan FP (CFP)-SNAP25-YFP, YsCsY) cleavage; in the absence of inhibitors, the RFU527/480 was approximately 1.2 at zero time, and was reduced to 0.8 upon incubation with BoNT/A-LC for 5 minutes. RFU527/480 values between 0.8 and 1.2 indicate a reduction in proteolytic activity (**Fig 3B)**. The results of both screens were consistent, and used to guide the selection of antibodies for further testing. Four mAbs that bound epitope I (9B2, 10B12, 10C9 and 11D8) inhibited proteolysis with statistical significance, p = 0.01, 0.004, 0.03 and 0.02 respectively using a one sample t test and results after 5 minutes of incubation. In the same epitope cluster scFv 1D9 1C7, 10B4 and 10H11 did not inhibit. scFv 1D2 (binding to epitope II) inhibited, but scFv 5A20.4 (binding to epitope IV) did not. scFv ING2 (binding to epitope III) inhibited but 12A11 did not.

The IC_50_ values of selected IgGs were measured using the FRET assay by fitting the initial catalytic rate and log [IgG] concentration to a sigmoidal dose-response model [39]. The results are summarized in Table 4. The IC_50_ of epitope cluster I mAbs 10B12, 10F9 and 11D8 were 7.38 × 10^−8^ M, 8.3 × 10^−9^ M 2.02 × 10^−8^ M respectively. Epitope cluster III mAbs 7C8 and ING2 also strongly inhibited proteolytic activity with IC_50_ of 1.05×10^−9^ M and 2.05×10^−9^ M.

**Table 4:** IC_50_ and K_D_ values of IgGs.

IgG	10B12^1^	10F9^1^	11D8^1^	7C8^2^	ING2^2^
IC_50_ (× 10−^9^ M) ^3^ (95% CI)	73.8 (11.86–41.34)	8.3 (7.55–91.12)	20.2 (6.08–73.07)	1.05 (0.08–0.87)	2.05 (0.47–5.71)
K_D._ (× 10−^9^ M) ^4^ (95% CI)	44.59 (<87.71)	9.81 (<24.92)	20.85 (13.99–30.11)	1.39 (1.18–1.62)	115.43 (93.62–134.99)

^1^ Measurements were performed in the FRET buffer.

^2^ Measurements were performed in the KGlu buffer.

^3^ Determined using FRET and YsCsY, performed in triplicate

^4^ Results of binding to BoNT/A LC from a single measurement using KinExA.

### Inhibition of SNAP25 cleavage in neuronal cells

**Fig 4:**
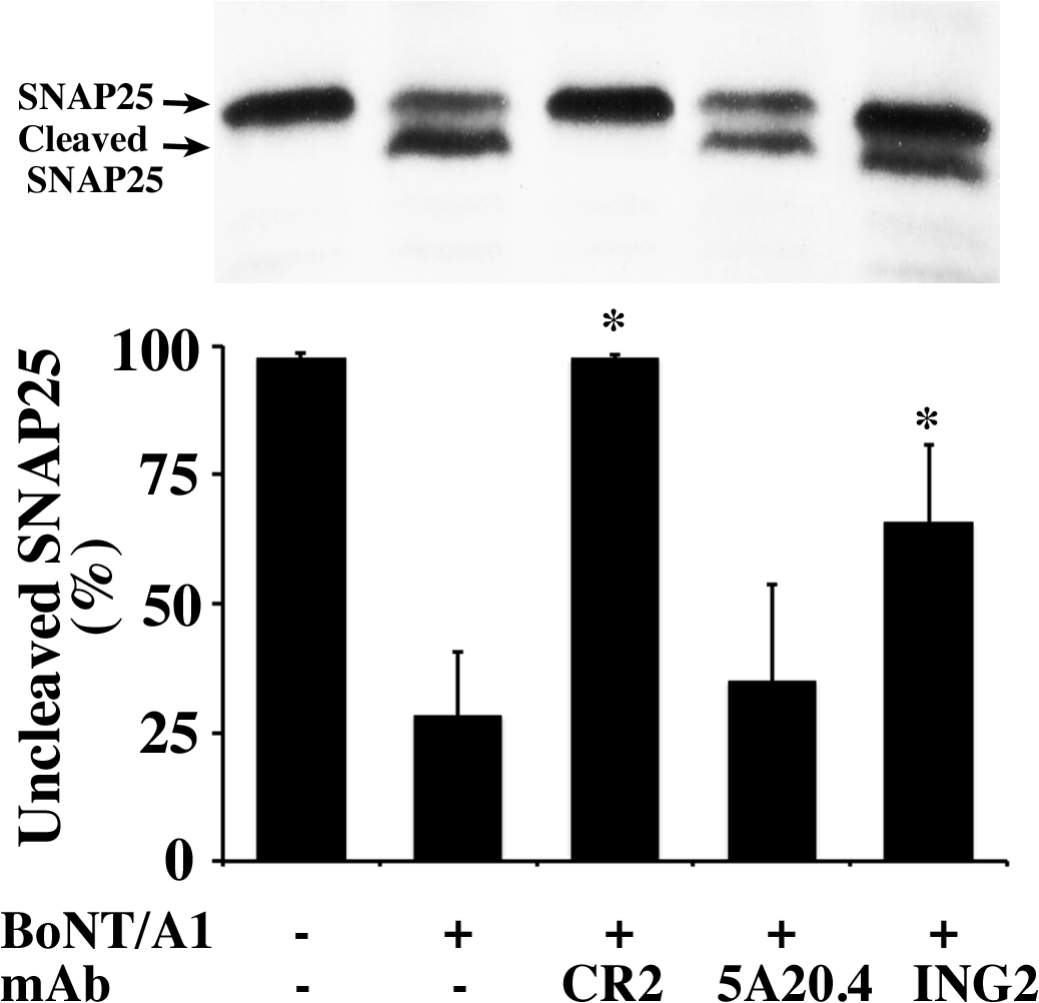
Inhibition of SNAP25 cleavage by mAbs in neuronal cells. Upper panel: Representative western blots (WB) of inhibition of BoNT/A-mediated SNAP25 cleavage by mAbs, indicating that 10 nM BoNT/A1 cleaved more than 50% of SNAP25 in Neuro-2a cells. Lower panel: Semi-quantitative analysis of the WB results. The experiments were repeated four times, and the percentage of non-cleaved SNAP25 was determined, shown as the mean ± SD. Compared with BoNT/A1 treated groups, CR2 (97.6 ± 0.6 vs. 28.2 ± 12.5, *P = 0.00003) and ING2 (65.7 ± 15.1 vs. 28.2 ± 12.5, *P = 0.0086) significantly reduced SNAP25 cleavage, while 5A20.4 did not (34.9 ± 18.9 vs. 28.2 ± 12.5, *P* = 0.58. Statistical significance was determined using the Holm-Sidak method for multiple t-tests with alpha = 5%, and no assumption of a consistent standard deviation using Graphpad Prism version 6.0 (La Jolla, CA).

The ability of selected mAbs to inhibit SNAP25 cleavage in neuronal cells was studied using the murine cholinergic neuroblastoma cell line Neuro-2a. mAbs studied included ING2, one of the two mAbs binding epitope III and inhibiting BoNT/A LC cleavage of SNAP-25 and 5A20.4, a non-inhibitory mAb binding epitope IV. We also evaluated mAb CR2, a BoNT/A H_C_ mAb previously shown to block BoNT/A uptake by neurons. We did not evaluate inhibitory Epitope 1 binding mAbs (10B12, 10F9 or 11D8) as they did not bind holotoxin and would not be expected to inhibit BoNT/A. Selected IgGs were added to cell cultures of the neuroblastoma cells in the presence of BoNT/A, and cleavage of intracellular SNAP25 was measured by Western blot analysis. In the absence of mAb inhibitors, 10nM BoNT/A cleaved over 50% of the cellular SNAP25 (Fig 4). mAb CR2, a BoNT/A H_C_-binding mAb that blocks neuronal uptake of BoNT significantly decreased SNAP-25 cleavage (p < 0.00003). The BoNT/A LC inhibitory mAb ING2 also decreased SNAP-25 cleavage (p<0.009) while the non-inhibitory mAb 5A20.4 did not (Fig 4).

### Fine Epitope mapping

To elucidate the epitopes associated with mAb-mediated inhibition of BoNT/A LC activity, the fine epitopes of seven mAbs were mapped by alanine-scanning mutagenesis using flow cytometry [45,46]. The mAbs selected included mAbs from each epitope cluster; 10F9 and 10B4, cluster I; 1D2, cluster II; 7C8, 12A11 and ING2, cluster III; and 5A20.4, cluster 4. To identify the general region where each mAb bound, 93 amino acids on the surface of BoNT/A LC were individually mutated to alanine (S1 Table and S1 Fig), displayed on yeast, and binding of each mAb at high concentration was determined. Mutants with loss or reduction of mAb binding were considered as important for binding. Unique mAb specific mutations that eliminated or significantly reduced binding were identified for mAbs 10F9, 10B4 and 5A20.4 (S2 Table). Two mutations (T122A and D141A) eliminated binding of all mAbs, while two mutations (F3A and K6A) eliminated binding of three mAbs (7C8, 12A11 and ING2) whose epitopes overlapped.

To determine the epitope of mAb 1D2 and better define the epitope of mAbs 7C8, 12A11 and ING2, a library of BoNT/A LC (size of 6×10^7^) with random mutations was constructed by using error prone PCR and selected using flow cytometry for loss of mAb binding [45]. For mAbs 7C8, and 12A11 and ING2, sequence analysis of clones with reduced binding showed amino acids in common or near each other when modeled on the BoNT/A crystal structure (see S3 Table for mAb 7C8). mAb 12A11 gave similar results to those of 7C8, while BoNT/A LC residues reducing mAb ING2 binding included V17, D18 and N514. For mAb 1D2, sequencing results indicated that clones with loss of binding had mutations between C430 and T442 or had a stop codon or reading frameshift before C430 (S4 Table) indicating that the epitope of 1D2 was located near the C-terminus of the BoNT/A LC. To confirm this, we constructed BoNT/A LC-H_N_, BoNT/A LC 1–380, 1–400, 1–425 and 390–448 that were displayed on yeast. Flow cytometry analysis showed 1D2 bound LC 1–448, LC 390–448 and LC-H_N_ but not LC1-380, 1–400, 1–425 or the holotoxins BoNT/A1 and A2 (S2 Fig). These results provided evidence that 1D2 bound amino acids between 425 and 448 of BoNT/A LC. The amino acids 438–447 are proteolytically cleaved by BoNT/A proteolytic processing [47] explaining why mAb 1D2 bound recombinant BoNT/A LC (1–448) and recombinant LC-H_N_ but not BoNT/A holotoxin.

To better map the mAb fine epitopes, 15–39 amino acids located around the amino acids identified by the above studies on the BoNT/A LC were mutated to alanine and each of these was expressed on the surface of yeast (S5 Table). The Fab of each mAb was prepared by digestion of IgGs with papain. The K_D_ values of Fab prepared from the IgG were measured for each yeast-displayed alanine mutant and wild-type BoNT/A LC using flow cytometry, and used to calculate the ΔΔG to determine the contribution of each amino acid to binding (S6 Table) [45]. The epitopes of mAbs 10F9 and 10B4 were modeled on the structure of the BoNT/A LC–SNAP25 complex (pdb ID, 1XTG) and the other mAbs that had epitopes on the LC near or including the H_N_ belt, on the structure BoNT/A surface (pdb ID, 3BTA).

**Fig 5:**
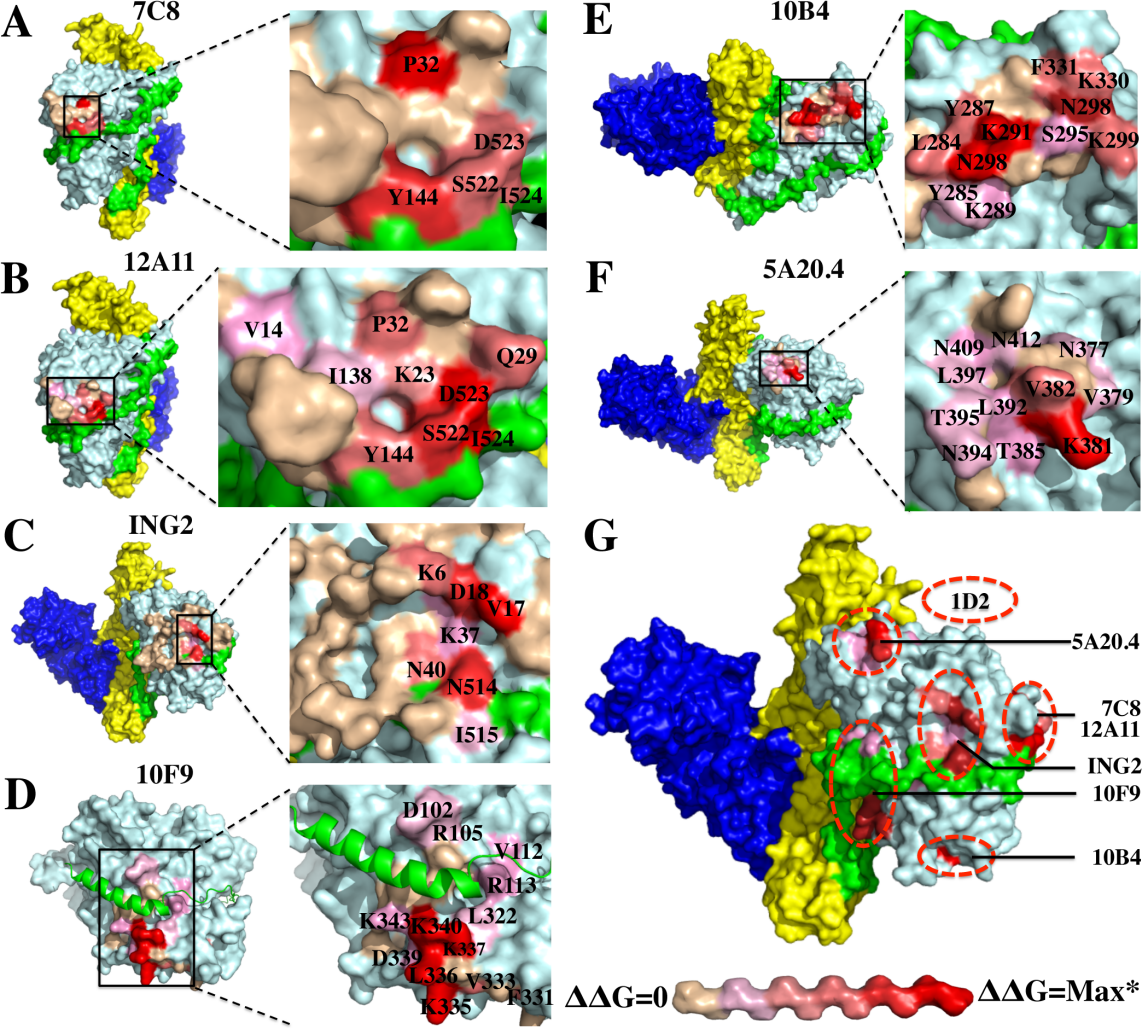
Fine epitope mapping of BoNT/A LC mAbs by alanine-scanning mutagenesis. (**A-F)** Amino acids near the mAb-binding sites on BoNT/A LC for representative mAbs (10F9, 10B4, and 5A20.4) or BoNT/A LC-H_N_ for representative mAbs (7C8, 12A11 and ING2) were mutated individually to alanine, and displayed on yeast (S2 Table). The K_D_ values of each mAb binding to the mutants and the wild type fragment were determined in triplicate. Epitopes modeled on the surface of the crystal structure of BoNT/A (pdb ID 3BTA) for 7C8, 12A11, ING2 and 5A20.4, or BoNT/A LC–SNAP25 complex (pdb ID 1XTG) for 10F9 and 10B4 using Pymol software. The ΔΔG for each pairing was then determined to evaluate the contribution of each amino acid in the epitope. (**G**) Maps of each of the epitopes on the surface of the BoNT/A LC structure. The differential contributions of amino acids in the epitope were colored using a gradient from red (denoting the greatest ΔΔG) to beige (denoting the smallest ΔΔG). *the maximum ΔΔG of each mAb as shown in S6 Table (7C8, ΔΔG*max* = 1.0; 12A11, ΔΔG*max* = 1.7; ING2, ΔΔG*max* = 1.9; 10F9, ΔΔG*max* >3.5; 10B4, ΔΔG*max* = 3.6; 5A20.4, ΔΔG*max* = 3.4).

As shown in Fig 5, 7C8, 12A11 and ING2 bound to amino acids on both the BoNT/A LC and the belt, consistent with the observation that they bound the holotoxin with much higher affinity than the isolated LC domain. The location of the epitopes of these three mAbs is consistent with the data showing that their epitopes overlap and that these mAbs cannot bind to antigen simultaneously. The epitope of 7C8 was located much closer to that of 12A11 compared to ING2, with the difference that the energetic interactions of 12A11 with belt amino acids was greater than for 7C8 (S6 Table, residues S522, D523, I524; Figs 5A and 4B). This difference explains the relative affinities of these two mAb for holotoxin compared to the affinities for LC (Tables 2 and 3). The epitope of ING2 was near that of 7C8 and 12A11 (V17, D18, N514; Fig 5C and 5G) and thus competed with 7C8 and 12A11 for BoNT/A LC binding. In a larger area on the BoNT/A LC, at least 14 amino acids made energetic contributions to the epitope of 10F9, located in the LC α1-helix (D102, R105, V112, R113) and the LC α3-helix (V333, K335, L336, K337, K340, K343) that comprise the α-exosite together with two other helixes (Fig 5D). Based on the ΔΔG values, the amino acids in the LC α3-helix contributed much more to the epitope than that in α1-helix (S3 Table). Finally, mAbs 10B4 and 5A20.4 bound BoNT/A LC distant from the substrate and belt-binding clefts and at non-overlapping sites; 10B4 had energetic interactions primarily with amino acids N288, K291 and K298 (Fig 5E), a location near enough to the epitope of mAb 10F9 to explain the inability of these two mAbs to bind antigen simultaneously; mAb 5A20.4 had energetic interactions with K381 and V382 (Fig 5F **)**.

Three mAbs (7C8, 12A11 and ING2) bound to both BoNT/A LC and the belt. The epitope of 7C8 was close to that of 12A11 on BoNT/A surface, with the difference that with 12A11 the residues in the belt region (S522, D523, I534) were more important for binding (panel A), while with 7C8 the LC residues (P32, Y114) were more important (panel B). The epitope of ING2, which competed with 7C8 and 12A11 for BoNT/A LC binding, was near that of the other two mAbs, but with different energetically important amino acids (V17, D18 on A-LC and N514 on the belt; panels C and G). The 10F9 mAb epitope included a large area on the BoNT/A LC surface involving amino acids both in the α1-helix (D102, R105, V112, R113, L322) and α3-helix (V333, K335, L336, K337, K340, K343) of the α-exosite overriding the SNAP25-binding cleft (panel D**).** mAbs 10B4 and 5A20.4 bound BoNT/A LC distant from the belt-binding cleft; 10B4 primarily binds N288, K291 and K298 on the BoNT/A LC (panel E) and 5A20.4 binds K381 and V382 (panel F).

## Conclusions

A new yeast display vector was constructed to generate scFv antibodies from immune murine V_H_ and V_L_ gene repertoires. The vector was constructed to allow simple two step sequential cloning of the V-genes, minimizing the number of PCR reactions needed compared to PCR splicing of the V_H_ and V_L_ genes [41,48]. The vector contains a 20 amino acid linker between V_H_ and V_L_ that is non redundant with the 15 amino acid (G_4_S)_3_ linker between Aga2 and the scFv gene. This eliminates the potential for deletion of the V_H_ gene due to homologous recombination that we have observed when the scFv linker is also (G_4_S)_3_.

After construction of three V gene libraries from mice immunized with BoNT/A LC, LC-belt or LC-H_N_ a total of seventeen BoNT/A LC-specific mAbs were obtained. Two additional mAbs were obtained from libraries constructed from humans immunized with toxoid. Differences in numbers of mAbs identified is likely related to the differences in immunogen (LC vs. toxoided holotoxin) and source of immune B-cells (spleen for mice and peripheral blood for humans). The scFv mAbs bound to BoNT/A LC with high affinity. mAb epitopes grouped into four clusters based on competition for binding to antigen. These clusters could be parsed into at least seven distinct epitopes by fine epitope mapping. These results demonstrate the value of yeast display of immune antibody repertoires for generating panels of high affinity antibodies with diverse epitopes.

Approximately half of the mAbs in epitope cluster I, as represented by mAb 10F9, inhibited the cleavage of the LC substrate SNAP-25 with IC_50_s of 8.3–74 x 10^−9^ M. SNAP-25 binds to LC in the groove covered by the H_N_ belt in the holotoxin and has a remarkably long substrate binding site of 16 amino acids that comprise the C-terminal of SNAP25, making it quite unique compared to other zinc proteases [49]. The Zn^2+^-containing catalytic center of BoNT/A LC is buried deeply within a narrow channel with size of 120nm×150nm×350nm [8], making it generally inaccessible to proteins like mAbs with size greater than 10nm in diameter. In addition to the catalytic center, SNAP-25 binds two exosites, an α-exosite containing four α helixes (α1, amino acids 102–113, α2, amino acids 310–321, α3, amino acids 335–348 and α4, amino acids 351–358) and a β-exosite containing two loops (loop 250, amino acids 242–259 and loop 370, amino acids 359–370). Mutations of residues in the exosites significantly eliminated the endopeptidase activity [36]. Fine epitope mapping indicated that some of the epitope 1 inhibitory mAbs bound the BoNT/A-LC on or around the α-exosite regions. When 10F9 is bound to the LC, the α-helix of SNAP-25 cannot bind at the α-exosite site and thus cleavage is inhibited. A characteristic of most epitope I mAbs is that they either do not bind holotoxin, or bind it with lower affinity than for LC. The H_N_ belt in the holotoxin masks this epitope.

We previously reported a single-domain llama antibody (Aa1) that also bound to BoNT/A LC at the α-exosite and showed potent inhibition of the endopeptidase [39]. Like the epitope I mAbs described above, Aa1 does not bind BoNT/A holotoxin. Instead the co-crystal structure of Aa1 bound to BoNT/A LC shows a salt bridge to LC amino acid K340 and hydrogen bonding of the antibody fragment to LC amino acids D102, R105, R113, and K356. LC K340 is a critical residue for binding of mAb 10F9. Amino acids R105 and R113 make energetic contributions to the binding affinity. Thus not only do these two mAbs share an overlapping epitope but also interact with some of the same LC amino acid side chains.

A second group of mAbs that bind epitopes composed of amino acids on both the LC and the H_N_ belt (7C8 and ING2) inhibited BoNT/A LC cleavage of SNAP-25 in vitro. ING2 also neutralized BoNT/A as measured by a reduction in SNAP-25 cleavage in a neuronal cell assay. The epitopes of these mAbs occupy sites where the extended SNAP-25 substrate binds and thus the mAbs when bound to LC block substrate binding and cleavage. However, while both mAbs bind to holotoxin with high affinity, especially as IgG, the binding affinity for LC is 100-fold to 1000-fold less than for holotoxin, since the H_N_ amino acids that comprise the epitope are not present. This lower affinity and partial loss of epitope amino acids likely explains the less potent inhibition.

A single human mAb and multiple macaque mAbs inhibiting BoNT/A cleavage of SNAP-25 have been previously reported [50,51]. Of these, the only mAb whose fine epitope was determined was SEM95-C6, a macaque scFv-Fc that inhibited SNAP-25 cleavage with “weak activity” in vitro but did not neutralize BoNT/A in an ex-vivo hemidiaphragm assay [51] Of note, the authors also reported four potent BoNT/A LC inhibiting mAbs that bound holotoxin with high affinity K_D_ ~ 1 x 10^−9^ M but no K_D_ for LC was reported nor the epitopes reported, due to ‘security’ concerns. One wonders whether these mAbs bound epitopes like those of 7C8 and ING2 or perhaps bound at sites with an allosteric effect. Based on the results presented here and in Dong et al. [39], it is unlikely these mAbs bind in the α-exosite, since they bind holotoxin with high affinity.

The results described here have implications for development of small molecule botulinum neurotoxin antidotes. Existing antibody-based antitoxins cannot enter the neuron and therefore cannot neutralize intracellular toxin and reverse paralysis. Development of such antidotes has focused on small molecules that prevent the LC from cleaving SNAP-25. Such inhibitors typically mimic substrate and bind in or around the substrate cleavage pocket [34,35]. The results here with mAbs confirm our earlier studies with a single domain antibody that mAbs that bind to the α-exosite, or other sites within the extended substrate-binding groove can inhibit substrate cleavage. Such exosites have been targeted for inhibitor development to other toxins [37,38] and more recently for BoNT/A [52], and a refinement to this approach could be to string together small molecules binding at different sites to achieve avidity and better substrate inhibition [53]. Alternatively, recently described liposomal “delivery vehicles” could be used to deliver inhibitory single chain or single domain antibodies into motor neurons to inhibit BoNT and reverse paralysis [54,55]. While inhibitory BoNT/A peptides have been delivered intracellularly using liposomes [54], this approach has yet to be demonstrated using antibodies or antibody genes. Technical challenges that need to be addressed include ability to load the delivery vehicle, whether the antibody could escape the endosome, intracellular stability, and activity of the antibody.

## Materials and Methods

### Ethics statement

The USAMRIID Institutional Animal Care and Use Committee approved the animal care and use protocol to conduct the animal studies reported here. Research was conducted under an IACUC approved protocol in compliance with the Animal Welfare Act, PHS Policy, and other Federal statutes and regulations relating to animals and experiments involving animals. The facility where this research was conducted is accredited by the Association for Assessment and Accreditation of Laboratory Animal Care, International and adheres to principles stated in the Guide for the Care and Use of Laboratory Animals, National Research Council, 2011. The specific national regulations and guidelines to which this animal care and use protocol adheres are the following: (1) 7 United States Code, Sections 2131–2159, Chapter 54 “Animal Welfare Act”, and (Code of Federal Regulations, Chapter 1, Subchapter A, Parts 1–4 “Animal Welfare Regulations”, (2) Health Research Extension Act of 1985, Public Law 99–158 “Animals in Research” and the Public Health Service Policy in Humane Care and Use of Laboratory Animals, (3) Biosafety in Microbiological and Biomedical Laboratories, 5th Edition, National Institute of Health, Human and Health Services Publication (CDC). 21–112, (4) Army Regulation 40–33 “The Care and Use of Animals in DOD Research, Development, Test and Evaluation or Training Programs” and (5) DOD Instruction 3216.01 “Use of Animals in DOD Programs”. USAMRIID is accredited by AAALAC/I. This agency uses the publication titled “The Guide for the Care and Use of Laboratory Animals”, 8th Edition, Institute for Laboratory Animal Research, National Research Council, as a guideline for evaluation and accreditation of program and it is based on the actual national regulations and guidelines for animal care and use programs. The animals used in this study were euthanized using carbon dioxide gas following the AVMA Guidelines on Euthanasia prior to spleen removal.

The University of California, San Francisco Institutional Review Board approved the human use protocol used for the studies described here.

### Oligonucleotides for mouse library construction

The primers for site-directed mutagenesis were designed and synthesized according to the manual of the QuikChange Site-Directed Mutagenesis Kit (Agilent, Palo Alto, CA). The primers for human library construction were synthesized as described [41]. The primers for mouse libraries were synthesized as following, the Gap tail for yeast gap-repairing transformation is underlined:

1. Primers for murine V_H_ gene amplification;
  VH1Gap5 5’-gactatgcagctagcggtgccatggcagaggtgcagcttcaggagtcagg-3’
  VH2Gap5 5’-gactatgcagctagcggtgccatggcagatgtgcagcttcaggagtcrgg-3’
  VH3Gap5 5’-gactatgcagctagcggtgccatggcacaggtgcagctgaagsagtcagg-3’
  VH4Gap5 5’-gactatgcagctagcggtgccatggcagaggtycagctgcarcartctgg-3’
  VH5Gap5 5’- gactatgcagctagcggtgccatggcacaggtycarctgcagcagyctgg-3’
  VH7Gap5 5’- gactatgcagctagcggtgccatggcagargtgaagctggtggartctgg -3’
  VH8Gap5 5’-gactatgcagctagcggtgccatggcagaggttcagcttcagcagtctgg-3’
  VH10Gap5 5’-gactatgcagctagcggtgccatggcagaagtgcagctgktggagwctgg-3’
  VH11Gap5 5’-gactatgcagctagcggtgccatggcacagatccagttgctgcagtctgg-3’
  VH3&4&5&10 deg Gap5 5’-gactatgcagctagcggtgccatggcasargtbcarctgnwrsarhcwgg-3’
  VH1&2&8 deg Gap5 5’-gactatgcagctagcggtgccatggcacgakgtkcagcttcagsagtcdgg-3’
  JH1Gap3 5’-gttgagcctccggacttaaggtcgactgaggagacggtgaccgtggtccc-3’
  JH2Gap3 5’-gttgagcctccggacttaaggtcgactgaggagactgtgagagtggtgcc-3’
  JH3Gap3 5’-gttgagcctccggacttaaggtcgactgcagagacagtgaccagagtccc-3’
  JH4Gap3 5’-gttgagcctccggacttaaggtcgactgaggagacggtgactgaggttcc-3’
  JH1-4 deg Gap3 5’-gttgagcctccggacttaaggtcgactgmrgagacdgtgashrdrgtbcc-3’
2. Primers for murine V_K_ gene amplification;
  VK1Gap5 5’-ggagaaggtagtagtggatccgcgcgcgacattgtgatgwcacagtctc-3’
  VK2Gap5 5’-ggagaaggtagtagtggatccgcgcgcgatgttktgatgacccaaactcc-3’
  VK3Gap5 5’- ggagaaggtagtagtggatccgcgcgcgatattgtgatracbcaggcwgc-3’
  VK4Gap5 5’-ggagaaggtagtagtggatccgcgcgcgacattgtgctgacmcartctcc-3’
  VK5Gap5 5’-ggagaaggtagtagtggatccgcgcgcsaaawtgtkctcacccagtctcc-3’
  VK6Gap5 5’-ggagaaggtagtagtggatccgcgcgcgayatyvwgatgacmcagwctcc-3’
  VK7Gap5 5’-ggagaaggtagtagtggatccgcgcgccaaattgttctcacccagtctcc-3’
  VK8Gap5 5’-ggagaaggtagtagtggatccgcgcgctcattattgcaggtgcttgtggg-3’
  VKdegGap5 5’-ggagaaggtagtagtggatccgcgcgcbmhdwhnwkmwvdyncwddydss-3’
  JK1Gap3 5’-ggcttaccttcgaagggcccgcctgcggccgctttgatttccagcttggtgcctcc-3’
  JK2Gap3 5’-ggcttaccttcgaagggcccgcctgcggccgcttttatttccagcttggtcccccc-3’
  JK3Gap3 5’-ggcttaccttcgaagggcccgcctgcggccgcttttatttccagtctggtcccatc-3’
  JK4Gap3 5’-ggcttaccttcgaagggcccgcctgcggccgcttttatttccaactttgtccccga-3’
  JK5Gap3 5’-ggcttaccttcgaagggcccgcctgcggccgctttcagctccagcttggtcccagc-3’
  JKdegGap3 5’-ggcttaccttcgaagggcccgcctgcggccgctttbakytccaryytkgtccchbm -3’

### Strains, media, antibodies, and toxin

YPD medium was used for growth of *Saccharomyces cerevisiae* strain EBY100, selective growth dextrose casamino acids media (SD-CAA) for selection of pYD4 transformed EBY100 and selective growth galactose CAA media (SG-CAA), for induction of scFv expression on the surface of EBY100. *E*. *coli* DH5α was used for subcloning and preparation of plasmid DNA, BL21, for BoNT fragments and GST-fused SNAP25 (141–206) expression and *E*. *coli* TG1 cells, for scFv-displaying phage packaging and soluble scFv expression. Pure holotoxins BoNT/A1 and A2 were purchased from Metabiologics (Madison, WI). All BoNT IgGs were expressed in Chinese hamster ovary cell (CHO) cells, while the mouse anti-SV5 antibody was purified from hybridoma and labeled with AlexaFluo-488 or AlexaFluo-647 labeling kit (Invitrogen, Carlsbad, CA). All the secondary antibodies including PE or APC-conjugated goat anti human-Fc, goat anti-mouse Fc and goat anti-human Fab were purchased from Jackson ImmunoResearch Laboratories (West Grove, PA).

### Protein production

Expression and purification of both BoNT/A LC (1–449) and GST-fused SNAP25 (141–206) was done as previously reported [39]. Briefly, synthetic BoNT genes were cloned and expressed in BL21 cells. The BoNT domains were purified using hexahistidine tags on IMAC columns and GST-fused SNAP-25 was purified on a glutathione-Sepharose 4B column.

### Mouse immunization and spleen harvest

Mice were vaccinated three times at two week intervals with 5 or 15 μg/mouse of BoNT/A LC (1–449), BoNT/A LC-belt (1–552), or BoNT/A LC-H_N_ (1–861) in 150 mM saline with 0.2% Alhydrogel (Brenntag Biosector, Frederickssund, Denmark). Details on the production and characterization of the BoNT/A LC antigens can be found in Jensen et al. [56]. Antibody titers after the third vaccination ranged from 0.2–10 x 10^−5^. Spleens were removed from mice 3–5 days after their final vaccinations and processed to extract mRNA for scFv library production using RNAgents total RNA isolation system (Promega, Madison, WI). Human scFv libraries were prepared using material from volunteers that had been vaccinated with pentavalent botulinum A-E toxoid. Details of vaccinations and RNA processing for the production of scFv libraries are reported in Amersdorfer et al. [57].

### Preparation of phage-displayed scFv and soluble scFv

The phage-displayed scFvs for 19 BoNT/A-LC mAbs was generated as described [58]. Briefly, scFv genes were prepared by digestion of pYD4-scFv plasmids with NcoI/NotI and were subcloned into the phagemid vector pSyn2 (pHEN) [41], following transformation of TG1 cells. The transformed TG1 cells were cultured in 2×YT/ampicillin/glutamine to an OD600 of 0.5, and then helper phage was added to the culture with a ratio of helper phage/bacteria at 20:1, incubating at 37°C for 30 minutes in a water bath and then at 37°C for 30 min with shaking. The culture was collected by centrifuge and resuspended in 2×YT/ampicillin/kanamycin and grown with shaking overnight at 30°C. The supernatant was collected by centrifuge and 1/10 volume of polyethylene glycol solution was used for phage precipitation. The phage pallet was collected and re-suspended in phosphate buffered saline (PBS), removing the bacterial debris by repeat centrifuging. The phage solution was finally stored at -80°C. For soluble scFv, the scFv genes were cloned into the plasmid pSyn1 and by *Nco*I/*Not*I digestion following transformation of TG1 cells. The scFv antibodies were expressed in TG1 cells by induction with 0.5mM IPTG in 2×YT/ampicillin at 18°C overnight. Periplasmic proteins were extracted by osmotic shock and the hexahistidine tagged scFvs were purified by IMAC on Ni-NTA agarose.

### Yeast displayed scFv library construction and library sorting

Total RNA was isolated from spleens of mice immunized with BoNT/A fragment (LC, LC belt or LC-H_N_) or from blood of 12 healthy donors immunized with BoNT toxoids subtype A, B, C, D and E. The cDNA was synthesized by RT-PCR with oligo dT as the primer using a ThermoScript RT-PCR Kit (Invitrogen, Carlsbad, CA). The V_H_ and V_K_ gene repertoires were amplified by PCR with primers without gap tails and then amplified with gap-tailed primers, followed by gel purification. For library construction, V_K_ gene repertoires were first cloned into the plasmid pYD4 (digested by *Nco*I/*Bss*HII) leading to a pYD4-V_K_ library; then the gap tailed V_H_ genes were transformed into EBY100 together with the *Nco*I/*Sal*I digested pYD4-V_K_ libraries by using LiAC, as previously described [40]. For the human libraries, the V_H_ and V_K_ genes were amplified from cDNA and then linked in a separate reaction with a (G_4_S)_3_ linker to obtain full length scFv genes by splicing with overlap extension PCR as previously described [41]. The scFv gene was combined with NcoI/Not I digested pYD2 plasmid and used to transform EBY 100. The library size was determined by total colony counting after plating serially-diluted transformation mixture on SD-CAA plates. The scFv libraries were induced by culturing the yeast in SG-CAA media with 10% SD-CAA.

The libraries were incubated with 50 nM of BoNT/A LC at RT for 1 hour. All following washing and staining steps were performed at 4°C using ice-cold FACS buffer (phosphate-buffered saline (pH 7.4), 0.5% bovine serum albumin). Washed yeast cells were incubated with 2 μg/mL of ING2 and 5A20.4 mAbs (mouse scFv libraries) or CR2 and RAZ1 mAbs (human scFv libraries) [59] for 60 min., washed, and then incubated 1 μg/mL of PE-labeled goat anti-human Fc antibody (Jackson Immunogenetic) and 1 μg/mL Alexa-647-labelled anti-SV5 mAb. After washing, yeast cells were flow sorted on a FACSAria II and the population with BoNT/A LC binding was gated and collected. The collected yeast were cultured and induced for the next round of sorting. After three rounds of sorting with sequential reduction in concentration of antigen, the yeast cells collected from the last round of sorting were plated on SD-CAA and cultured at 30°C for 48 hours. Individual colonies were picked, grown, and induced in 96 deep-well plates, then screened for BoNT/A LC binding using the same staining conditions used for sorting. Unique BoNT/A LC binding clones were identified by DNA sequencing.

### K_D_ Measurement

The equilibrium dissociation constant (K_D_) of yeast displayed scFvs was measured by flow cytometry as previously described with modification [39,40]. Briefly, 1 x 10^6^ yeast displaying scFvs were incubated in FACS buffer with six different concentrations of BoNT/A LC or holotoxin that ranged at least 10 fold higher and 10 fold lower than the expected K_D_ at room temperature for 1 hour. Ice-cold FACS buffer was used to wash the samples and 2 μg/ml of ING2 was applied at 4°C for 60 minutes following 1 μg/ul of phycoerythrin (PE)-conjugated goat anti-human IgG and 1 μg/mL Alexa-647-labelled anti-SV5 mAb at 4°C for 30 minutes. Finally, the yeasts were washed by cold FACS buffer and the mean fluorescence (MFl) of BoNT/A LC binding was measured by flow cytometry. The MFI was plotted against the concentration of antigen and the K_D_ was determined by the following equation:

y=m1+m2*m0/(m3+m0),

where y = MFl at a given antigen concentration, m0 = Antigen concentration, m1 = MFI of the no antigen control, m2 = MFl at saturation, and m3 = K_D_.

For selected IgGs, the solution phase affinity at equilibrium and binding kinetics were measured using flow fluorimetry in a KinExA as previously described [39,40]. Briefly, IgG was serially diluted into a constant concentration of BoNT/A LC or BoNT/A holotoxin. After reaching equilibrium, samples were passed over a flow cell with a 4 mm column of Azlactone beads (Sapidyne Instruments) covalently coated with the same IgG to capture the free antigen molecules, which were quantitated by flowing Alexa-647 labeled secondary antibody over the beads. The equilibrium titration data were fit to a reversible binding model using GraphPad Prism Software to determine the K_D_.

### Epitope classification of mAbs

mAb were classified into epitope groups based on their ability to compete with each other for binding to antigen. Briefly, yeast-displayed scFv were incubated for 60 min with 25 nM of BoNT/A LC (residues 1–437) in solution and then the ability of scFvs displayed on the surface of phage to bind to the LC was determined by incubation for 60 min. with 100 μl of crude phage solution displaying the relevant scFv. Phage antibody binding was detected by incubation for 60 min. with 1 μg/mL of PE-conjugated anti-M13 antibody with 1 μg/mL of Alex-647 labeled SV5 antibody to detect yeast displayed scFv. Binding was measured by flow cytometry. Phage displayed scFv that bound an overlapping epitope to yeast displayed scFv showed no PE-signal, while those binding non-overlapping epitopes showed a positive PE signal. Binding of the same phage displayed scFv as the yeast-displayed scFv was used as a positive control for overlapping epitopes.

### MAb Inhibition of SNAP-25 cleavage by BoNT/A-LC

For SDS-based analysis of substrate cleavage, 25nM of BoNT/A LC and 500nM of scFv (20 times higher scFv than LC) were mixed in Tris buffer (50 mM Tris buffer, pH 8.0), and then 5 μM of GST-SNAP-25 (141–206) was added to initiate the reaction. Aliquots of reaction were extracted at 5 and 15 minutes and the reaction stopped by addition of SDS-PAGE loading buffer. Samples were heated for 10 min at 95°C in SDS-PAGE loading buffer and loaded on 15% SDS-PAGE Gel for electrophoresis and detected with Coomassie staining [39]. SDS-PAGE assay was repeated three times; a representative gel is shown in the results section. For FRET-based analysis, 2 μM of YsCsY was mixed with 200 nM mAbs in FRET buffer (20 mM HEPES, pH 7.5; 1.25mM DTT, 10 μM ZnCl_2_, 0.2% Tween20, 0.1 mg/ml BSA) in a black 96-well plate (Corning). After pre-incubating at 30 minutes, 400 pM BoNT/A LC was added. With excitation at 425 nm and with the cutoff at 495 nm, the emission at 527 nm and 480 nm were measured in a fluorescence reader (Spectra Max Gemini, Molecular Devices) after 5 minutes and 15 minutes of digestion. The ratio of Fluo527/Fluo480 was calculated for evaluation of YsCsY cleavage. The higher the ratio, the more undigested YsCsY is left in the reaction.

The 50% inhibitory concentration (IC_50_) of selected IgGs were measured using the method of Pires-Alves et al. [43]. For mAbs 10B12, 10F9 and 11D8, the assays were performed in the reported buffer (10 mM HEPES, 150 mM K glutamate, 0.01% Tween20, pH 7.2); for mAbs ING2 and 7C8, an alternate assay buffer (20 mM HEPES pH 7.5, 0.2% Tween, 10 μM ZnCl_2_, 0.1mg/ml BSA) was used since these mAbs did not inhibit BoNT/A LC in the HEPES/glutamate buffer. Briefly, 0.5 μM of YsCsY was first mixed with two-fold serially diluted IgG. After pre-incubation at 30°C for 15 min, 1 nM of BoNT/A-LC was added and the emission at 527 nm was measured at 5 seconds intervals. The rate of decay of YFP fluorescence (Fluo527) was equated to the cleavage rate of YsCsY. The initial cleavage rate (R) of the enzyme was calculated by fitting the Fluo527 emission for the first 40 seconds to a simple linear regression model: Y = RX + C, where Y = Fluo527, R = the initial rate (slope), X = time, and C = y-intercept. IC_50_ values were determined by fitting the initial rate and log IgG concentration to a sigmoidal dose-response (variable slope) model (GraphPad Prism version 6.0).

### Site-directed mutagenesis

Alanine mutants of BoNT/A LC (S5 Table) were prepared following the instruction manual of the QuikChange II-E Site-Directed Mutagenesis Kit (Agilent Tech, Palo Alto CA). Briefly, the primers containing the mutation were used for PCR amplification with the plasmid pYD4 containing the BoNT/A LC gene pYD4A-LC or containing the BoNT/A LC-H_N_ gene (pYD4-A-LC-H_N_) as a template for 18 cycles. The PCR product was digested with DpnI to remove the parental methylated and hemimethylated DNA, which was then purified by StrataClean Resin and transformed into *E*. *coli* XL1-Blue. The alanine mutants of BoNT/A LC or BoNT/A LCH_N_ in pYD4 were than individually transformed into EBY100, grown in SD-CAA and induced in SG-CAA for expression on the surface of EBY100. DNA sequencing was used to verify each construct.

### Library construction and sorting of random mutants of BoNT/A LC

A BoNT/A LC fragment library with random mutations was prepared by using error-prone PCR with the primers pYD-For/pYD-Rev and DNA polymerase Paq5000 (Agilent Tech) plus 12.5 μM MnCl_2_ [45]. The PCR product was then gel purified, and the fragment was inserted into the 30 NcoI/NotI sites of the plasmid pYD4, and the resulting ligation mixture transformed into EBY100. The library was cultured in SD-CAA for 48 hours, and then 50 ml of the culture was induced with galactose in 500 ml SG-CAA at 18°C for 48 hours. For 1D2 epitope mapping, the library was sorted by incubation with 1 μg/mL of 1D2 mAb and 1 μg/mL of ING2 mAb followed by incubation with 1 μg/mL of goat anti-mouse-PE (Jackson Immuno Research, West Grove, PA) and 1 μg/mL of goat-antihuman-PE (Jackson Immuno Research, West Grove, PA). Yeast binding both 1D2 and ING2 were collected for the 2^nd^ round of sorting, in which the library was again incubated with 1D2 with binding detected with goat-antimouse-PE and 1 μg/mL of ING2 conjugated to Alexa-647. For this sort only the ING2 binding yeast were collected. In the 3^rd^ round of sorting, the enriched library was incubated with ING2-Alexa647 and 1D2 followed by goat-anti-mouse-PE and 1 μg/mL of SV5 mAb conjugated to Alexa488. The population binding ING2 but not binding 1D2 was again collected and plated on SD-CAA plate for individual colony analysis. The mutants with loss of binding to 1D2 were considered as amino acids in the 1D2 epitope. Using the same approach, the critical amino acids for the ING2, 7C8 and 12A11 epitopes were defined.

### Preparation of Fab from IgG Fab

Fab fragments were generated by digestion of purified IgG with papain (Pierce Biotechnology, IL). Briefly, ∼12 mg/ml IgG in 20 mM phosphate (pH 7.0) with10 mM EDTA was incubated with an equal volume of immobilized papain resin at 37°C for 16 h. The immobilized papain resin was removed by centrifugation, and the digest supernatant was dialyzed against 10 mM MES (pH 5.6). The Fab was separated from undigested IgG and Fc fragments by cation-exchange chromatography (HiTrap SP HP, GE Healthcare, NJ) using a salt gradient. The purified Fab was then dialyzed against PBS and stored at −80°C.

### Fine epitope mapping of selected mAbs

BoNT/A LC residues that were energetically important for mAb binding were determined by comparing the change of Gibbs free energy (ΔΔG) for alanine mutants of BoNT/A LC (or BoNT/A LC-H_N_) with that for the wild-type fragment [45]. Using 7C8 as an example, the residues located near the putative epitope (identified by the studies described above) were mutated to alanine by site-directed mutagenesis and displayed on the surface of yeast. Using serial dilutions of 7C8 Fab, the K_D_ value for 7C8 binding to each of the yeast-displayed BoNT/A LC-H_N_ mutants, compared to wild type, was determined. All the K_D_ values were determined in triplicate (S6 Table). The ΔΔGs for mAbs were calculated to evaluate the contribution of each amino acid in the epitope using the following formula:

$$\Delta \Delta \text{G}\;\left(\text{Kcal/mol}\right)={\text{RT}}^{*}\text{ln}\left({\text{K}}_{\text{D}}{\text{-Mut/ K}}_{\text{D}}\text{-Wt}\right)/1000,$$

where R = 1.985 8775 Cal/K mol; T (K) = 20 (°C) + 223.15. Using the ΔΔG values, molecular models of the epitopes were constructed, as shown in Fig 5.

### Neuronal Cell assays

The murine cholinergic neuroblastoma cell line Neuro-2a (CCL-131; ATCC, Manassas, VA) was used for assay of inhibitory activity of BoNT/A LC mAbs *in vitro*. Briefly, Neuro-2a cells were cultured in 12-well culture plates in Opti-MEM containing 10% FBS, glutamine and penicillin /streptomycin (Invitrogen). After 48 hours, the medium was replaced with Opti-MEM, containing 2% FBS and cells were incubated for an additional 3 hours at 37°C. Cells were then treated with the mixture of BoNT/A1 (final concentration of 50 nM) and mAbs (final concentration of 10 nM) together with 100 μg/ml of Trisialoganglioside-GT1b (Matreya LLC, State College PA). Cells without mAb treatment and without BoNT/A were used as controls. After 30 minutes of incubation, the media was removed and cells were scraped off the dishes and protein was extracted by using RIPA lysis and extraction buffer with proteinase inhibitors (Thermo Scientific). After centrifugation at 12,000 rpm, for 20 minutes, the supernatant were collected and protein concentration was determined by the absorbance at the 280 nm.

An equal amount of protein (40 μg) was loaded onto a 4–20% SDS-PAGE gradient gel for electrophoresis. Subsequently, proteins were transferred onto a polyvinylidene difluoride membrane (iBlot gel transfer device, Life Technologies). After blocking with 5% fat-free milk, the membrane was immunoblotted overnight with a primary antibody against SNAP-25 (Santa Cruz Biotechnology Inc., CA) at 4°C, followed by incubation with horseradish peroxidase-conjugated anti-mouse secondary antibody (Jackson Immuno Research, West Grove, PA) and visualization using SuperSignal West Femto kit (Thermo Scientific, Grand Island, NY) and autoradiography using Kodak film (Kodak Co., Rochester, NY). The scanned images were analyzed by using NIH Image J software for semi-quantitation. The percentage of non-cleaved SNAP-25 was determined to assess the mAb inhibitory activity of BoNT/A LC.

## Supporting Information

S1 Fig Distribution of 93 individual alanine mutants on the BoNT/A LC surface used for mAb epitope mapping. (TIF)

S2 Fig FACS analysis of 1D2 binding to BoNT/A toxins and fragments. **A.** Lack of binding of 1D2 to BoNT/A holotoxin. Yeast displayed 1D2 scFv was incubated with holotoxin and then with anti-BoNT/A mAb RAZ1 to detect holotoxin binding. 1D2 did not bind either BoNT/A1 or A2 holotoxins, which do not include amino acids 438–447. **B.** Identification of 1D2 epitope: To determine the epitope of 1D2, BoNT/A-LC-HN, full length BoNT/A LC (1–448) and truncated BoNT/A LC (1–380, 1–400, 1–425, and 390–448) were displayed on the yeast surface. FACS analysis indicated that 1D2 did not bind BoNT/A LC (1–380), (1–400) or (1–425) but bound BoNT/A LC (390–448) and BoNT/A LC-HN. This indicates that 1D2 has a linear epitope at the C-terminus of full length BoNT/A LC (amino acids 425–448). The experiments were conducted in triplicate.
(TIF)

S1 Table Mutants on the BoNT/A LC surface changed to alanine for mAb epitope mapping. List of alanine mutants on the surface to BoNT/ A that were made for epitope mapping.
(PDF)

S2 Table BoNT/A LC mutants that eliminated mAb binding. List of BoNT/A LC mutants that eliminated mutants for listed mAbs.
(PDF)

S3 Table BoNT/A-LC mutants that eliminated 7C8 binding. List of mutants that eliminated binding ofr the mAb 7C8
(PDF)

S4 Table BoNT/A LC mutants that eliminated mAb 1D2 binding. List of mutants that eliminated binding ofr the mAb 1D2
(PDF)

S5 Table BoNT/A LC or BoNT/ A LCH_N_ alanine mutants used for mAb fine epitope mapping. List of alanine mutants used for fine epitope mapping of various antibodies.
(PDF)

S6 Table K_D_ and ΔΔG of selected BoNT/A LC alanine mutants for fine epitope mapping. K_D_ and ΔΔG values of selected mutants of BoNT/A that were used to conduct fine epitope mapping.
(PDF)
