# Supplementary figures and images for: Monoclonal Antibodies Targeting the Alpha-Exosite of Botulinum Neurotoxin Serotype/A Inhibit Catalytic Activity
Source: PLoS One. 2015 Aug 14;10(8):e0135306. doi: 10.1371/journal.pone.0135306 (PMC4537209; doi:10.1371/journal.pone.0135306)

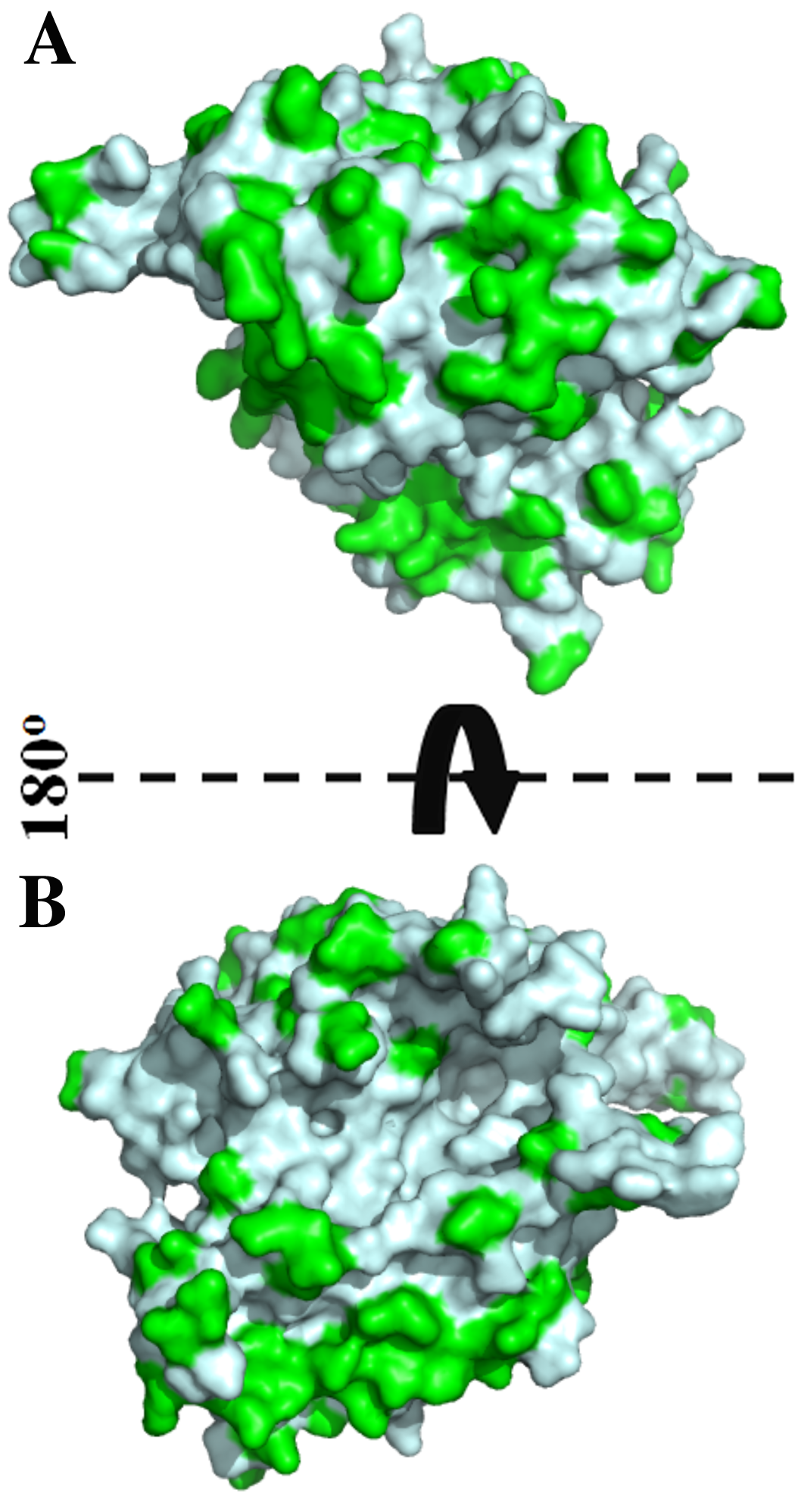

Supplement: S1 Fig — (TIF) [file pone.0135306.s001.tif]

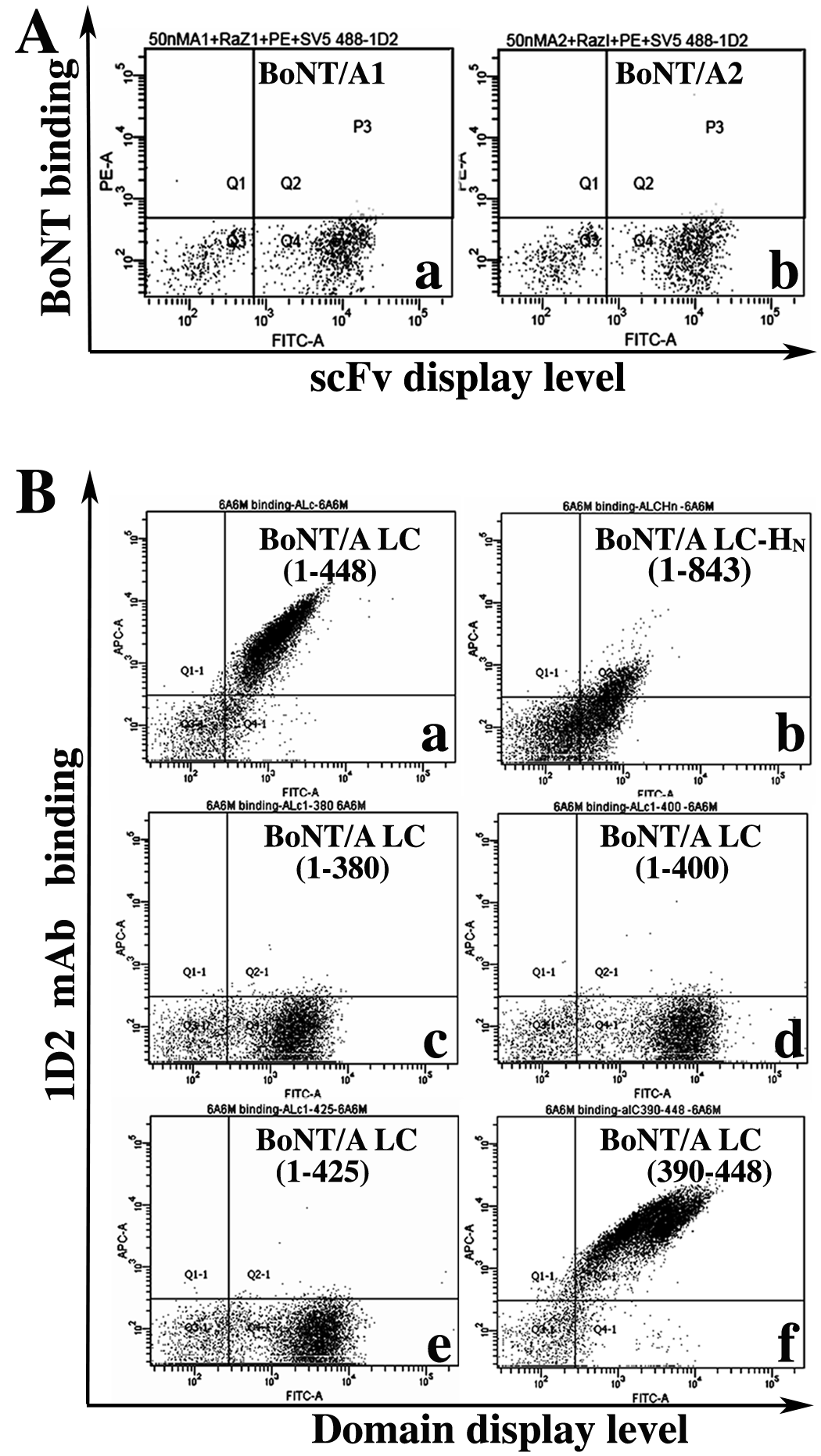

Supplement: S2 Fig — A. Lack of binding of 1D2 to BoNT/A holotoxin. Yeast displayed 1D2 scFv was incubated with holotoxin and then with anti-BoNT/A mAb RAZ1 to detect holotoxin binding. 1D2 did not bind either BoNT/A1 or A2 holotoxins, which do not include amino acids 438–447. B. Identification of 1D2 epitope: To determine the epitope of 1D2, BoNT/A-LC-HN, full length BoNT/A LC (1–448) and truncated BoNT/A LC (1–380, 1–400, 1–425, and 390–448) were displayed on the yeast surface. FACS analysis indicated that 1D2 did not bind BoNT/A LC (1–380), (1–400) or (1–425) but bound BoNT/A LC (390–448) and BoNT/A LC-HN. This indicates that 1D2 has a linear epitope at the C-terminus of full length BoNT/A LC (amino acids 425–448). The experiments were conducted in triplicate. (TIF) [file pone.0135306.s002.tif]
