# Supplementary material for: Monoclonal Antibodies Targeting the Alpha-Exosite of Botulinum Neurotoxin Serotype/A Inhibit Catalytic Activity
Source: PLoS One. 2015 Aug 14;10(8):e0135306. doi: 10.1371/journal.pone.0135306 (PMC4537209; doi:10.1371/journal.pone.0135306)
Supplement: S1 Table — List of alanine mutants on the surface to BoNT/ A that were made for epitope mapping. (PDF) [file pone.0135306.s003.pdf]

Table S1. Mutants on the BoNT/A LC surface changed to alanine for mAb epitope mapping.

F3A	N5A	K6A	Q7A	N9A	K11A	P13A	Q29A
N40A	K41A	E47A	T50A	E55A	D58A	N60A	P63A
T76A	S79A	K89A	T92A	S100A	D102A	T122A	I123A
K128A	D131A	Q139A	D141A	Q162A	E171A	V172A	L173A
D203A	L207A	K212A	N240A	K244A	N246A	E257A	H269A
K272A	Q278A	E279A	N280A	E281A	R283A	L284A	Y285A
N288A	K289A	K291A	D292A	S295A	T296A	K299A	K301A
V304A	S309A	Y312A	K314A	K318A	E319A	L322A	T327A
K330A	S332A	D334A	K337A	D339A	K340A	L341A	E347A
D352A	K356A	F357A	K359A	K371A	K375A	N377A	K381A
V382A	Y387A	R393A	N394A	T395A	L397A	E407A	M411A
K415A	K417A	E424A	L428A	R432A			
