# Supplementary material for: Monoclonal Antibodies Targeting the Alpha-Exosite of Botulinum Neurotoxin Serotype/A Inhibit Catalytic Activity
Source: PLoS One. 2015 Aug 14;10(8):e0135306. doi: 10.1371/journal.pone.0135306 (PMC4537209; doi:10.1371/journal.pone.0135306)
Supplement: S2 Table — List of BoNT/A LC mutants that eliminated mutants for listed mAbs. (PDF) [file pone.0135306.s004.pdf]

Table S2. BoNT/A LC mutants that eliminated mAb binding

mAb	Mutants that eliminate mAb binding
**7C8**	F3A; K6A; T122A; D141A
**10F9**	K291A, K337A, D339A, K340A, R283A; T122A; D141A
**12A11**	F3A; K6A; T122A; D141A
**1D2**	T122A; D141A
**10B4**	L284A, N288A, K291A, L341A; T122A; D141A
**ING2**	F3A; K6A; T122A; D141A
**5A20.4**	K381A; V382A; T122A; D141A
