# Supplementary material for: Monoclonal Antibodies Targeting the Alpha-Exosite of Botulinum Neurotoxin Serotype/A Inhibit Catalytic Activity
Source: PLoS One. 2015 Aug 14;10(8):e0135306. doi: 10.1371/journal.pone.0135306 (PMC4537209; doi:10.1371/journal.pone.0135306)
Supplement: S3 Table — List of mutants that eliminated binding ofr the mAb 7C8 (PDF) [file pone.0135306.s005.pdf]

Table S3. BoNT/A-LC mutants that eliminated 7C8 binding

Colony	Mutation
1	**Q31K** M106T S110Y A228T
2	**P32A** E257Q
3	P**32A** G169S G255D E347D
4	V14I **P32A**
5	**Q31A** E64V H230R
6	**Q31H I138F** F194Y G211D R264H E351Q
7	**Q31R** E83D W118S T215A K364N
8	**Q31R** E83D W118S T215A
9	**Q31R** F213I T307N
10	**Q31R** S71F M106T F213I
11	**P32S** N53S E262D
12	**P32S**
13	**Y21C** A308T
14	**I138T** F331L
15	**I138F** T176S N240K
16	D85K **I138T** N246A

Bolded mutants are those that occur in multiple clones or are near residues that occur in multiple clones.
