# Supplementary material for: Monoclonal Antibodies Targeting the Alpha-Exosite of Botulinum Neurotoxin Serotype/A Inhibit Catalytic Activity
Source: PLoS One. 2015 Aug 14;10(8):e0135306. doi: 10.1371/journal.pone.0135306 (PMC4537209; doi:10.1371/journal.pone.0135306)
Supplement: S4 Table — List of mutants that eliminated binding ofr the mAb 1D2 (PDF) [file pone.0135306.s006.pdf]

Table S4. BoNT/A LC mutants that eliminated mAb 1D2 binding

Colony	Mutation
**A2**	S254F, F260V, K340N, K375I, T385S, **T436I**
**A4**	No mutation, **stop at Y250**
**A9**	P206S, T350N, F390C, **shift at N409**
**C7**	F95I, A249T, T215I, **L442Q**
**C9**	F192L, A308T, V219I, V382D, I435N, **T436I**
**E5**	A158T, I293M, K343N, **T439N**
**F1**	P62S, F290Y, **Stop at C430**
**F12**	M30K R363S, V129L, Y251H, **T436I**
**G8**	G8: Q67H, S71F, A222V, D275V, A372T, **T436N**

Bolded mutants are those that occur in multiple clones or are near residues that occur in multiple clones.
