# Supplementary material for: Monoclonal Antibodies Targeting the Alpha-Exosite of Botulinum Neurotoxin Serotype/A Inhibit Catalytic Activity
Source: PLoS One. 2015 Aug 14;10(8):e0135306. doi: 10.1371/journal.pone.0135306 (PMC4537209; doi:10.1371/journal.pone.0135306)
Supplement: S5 Table — List of alanine mutants used for fine epitope mapping of various antibodies. (PDF) [file pone.0135306.s007.pdf]

Table S5. BoNT/A LC or BoNT/ A LCH$_N$ alanine mutants used for mAb fine epitope mapping.

Antibody	**BoNT/A-LC Mutants used for epitope mapping**
7C8<br>(A LC-H$_N$)	P13A; V14A; K23A; Q29A; M30A; Q31A; P32A; V33A; K34A; I138A; Q139A; P140A; D141A; G142A; S143A; Y144A; E147A; R145A; E513A; S522A; D523A; I524A
10F9<br>(A LC)	E279A; F282A; L322A; K330A; F331A; S332A; V333A; D334A; K335A; L336A; K337A; D339A; K340A; L341A; K343A; M344A; E347A; I348A; R105A; M106A; D102A; L103A; S110A; V112A; R113A
12A11<br>(A LC-H$_N$)	P13A;V14A;K23A;M30A;Q31A; V33A; K34A; I138A;D141A; Y144A; R145A; E513A; S522A; D523A; I524A; Q29A; P32A; Q139A; P140A; G142A; S143A; E147A
10B4<br>(A LC)	N280A; E281A; F282A; L284A; Y285A; Y286A; Y287A; N288A; K289A; F290A; K291A; D292A; S295A; N298A; K299A; K330A; F331A; S332A; V333A
ING2<br>(A LC-H$_N$)	F3A; N5A; K6A; Q7A; F8A; N9A; D12A; P13A; V17A;D18A; K37A; H39A; N40A; E96A; Y99A; R105A; M106A; Q139A; P140A; D141A; G142A; S143A; Y144A; R145A; E147A; 386A; Y387A; Y503A; L504A; T505A; F506A; N507A; 508A;D509A; N510A; E511A; E513A; N514A; I515A; E513A
5A20.4<br>(A LC)	N377A; V379A; P380A; K381A; V382A; N383A; T385A; L392A;R393A; N394A; T395A; L397A; N409A; M411A; N412A.
