# Supplementary material for: Monoclonal Antibodies Targeting the Alpha-Exosite of Botulinum Neurotoxin Serotype/A Inhibit Catalytic Activity
Source: PLoS One. 2015 Aug 14;10(8):e0135306. doi: 10.1371/journal.pone.0135306 (PMC4537209; doi:10.1371/journal.pone.0135306)
Supplement: S6 Table — KD and ΔΔG values of selected mutants of BoNT/A that were used to conduct fine epitope mapping. (PDF) [file pone.0135306.s008.pdf]

Table S6. K_D and ΔΔG of selected BoNT/A LC alanine mutants for fine epitope mapping.

7C8				12A11				ING2			
Mutant	K_D (pM)	Ratio K_D Mt/Wt	ΔΔG (kcal/mol)	Mutant	K_D (pM)	Ratio K_D Mt/Wt	ΔΔG (kcal/mol)	Mutant	K_D (pM)	Ratio K_D Mt/Wt	ΔΔG (kcal/mol)
Y144A	410.8	3.80	0.78	V14A	289.5	2.32	0.49	K6A	558.3	3.69	0.76
S522A	291.1	2.69	0.58	K23A	384.1	3.09	0.66	V17A	3654	24.2	1.85
D523A	293.2	2.71	0.58	Q29A	551.6	4.44	0.87	D18A	2377	15.7	1.61
I524A	168.3	3.41	0.71	P32A	428.6	3.45	0.72	K37A	361.0	2.39	0.51
P32A	529.0	4.90	0.92	I138A	210.9	1.69	0.31	N40A	645.1	4.27	0.85
A LC-H_N	108.0	-	-	Y144A	451.4	3.63	0.75	N514A	2165	14.3	1.55
				S522A	843.1	6.78	1.11	I515A	161.9	1.07	0.04
				D523A	2132	17.2	1.65	E147A	340.4	2.25	0.47
				I524A	660.4	5.32	0.97	A-LCH_N	151.3	/	/
				A-LCH_N	124.2	-	/				

10F9				10B4				5A20.4			
Mutant	K_D (pM)	Ratio K_D Mt/Wt	ΔΔG (kcal/mol)	Mutant	K_D (pM)	Ratio K_D Mt/Wt	ΔΔG (kcal/mol)	Mutant	K_D (pM)	Ratio K_D Mt/Wt	ΔΔG (kcal/mol)
L322A	235.2	1.59	0.27	F282A	7.05	2.32	0.49	N377A	207.9	1.55	0.25
F331A	1683	11.4	1.42	L284A	165.4	54.5	2.33	V379A	212.8	1.58	0.27
V333A	3901	26.4	1.91	Y285A	6.59	2.17	0.45	K381A	41823	311	3.34
K335A	>>	-	>3.5	Y286A	5.82	1.92	0.38	V382A	1999	14.9	1.57
L336A	>>	-	>3.5	Y287A	82.78	27.3	1.92	T385A	340.7	2.53	0.54
K337A	>>	-	>3.5	N288A	253.8	83.6	2.58	L392A	508.1	3.78	0.77
D339A	363.3	2.46	0.52	K289A	4.72	1.56	0.26	N394A	197.8	1.47	0.23
K340A	>>		>3.5	F290A	153.6	50.3	2.28	T395A	196.7	1.46	0.22
L341A	6004	40.6	2.16	K291A	1399	461	3.57	L397A	201.2	1.49	0.24
K343A	398.9	2.70	0.58	S295A	6.70	2.20	0.46	N409A	275.0	2.05	0.42
A-LC*	160.5	-	-	N298A	413.9	136	2.86	N412A	208.6	1.55	0.26

R105A	118.0	2.21	0.46	K299A	60.92	20.1	1.75	A-LC	134.4	/	/
D102A	108.8	2.04	0.42	K330A	20.42	6.72	1.11				
V112A	182.1	3.41	0.71	F331A	46.55	15.3	1.59				
R113A*	129.5	2.43	0.52	A-LC	3.04	/	/				
A-LC	53.4	-	-								

* The KDs of the mutants were measured in two different batches of the wide type A-LC.
